# Role of Chrononutrition in the Antihypertensive Effects of Natural Bioactive Compounds

**DOI:** 10.3390/nu14091920

**Published:** 2022-05-04

**Authors:** Néstor Ibarz-Blanch, Diego Morales, Enrique Calvo, Laura Ros-Medina, Begoña Muguerza, Francisca Isabel Bravo, Manuel Suárez

**Affiliations:** Nutrigenomics Research Group, Departament de Bioquímica i Biotecnologia, Universitat Rovira i Virgili, 43007 Tarragona, Spain; nestor.ibarz@urv.cat (N.I.-B.); diego.morales@urv.cat (D.M.); laurarosmedina@gmail.com (L.R.-M.); begona.muguerza@urv.cat (B.M.); manuel.suarez@urv.cat (M.S.)

**Keywords:** blood pressure, biological rhythms, hypertension, peptides, phenolic compounds

## Abstract

Hypertension (HTN) is one of the main cardiovascular risk factors and is considered a major public health problem. Numerous approaches have been developed to lower blood pressure (BP) in hypertensive patients, most of them involving pharmacological treatments. Within this context, natural bioactive compounds have emerged as a promising alternative to drugs in HTN prevention. This work reviews not only the mechanisms of BP regulation by these antihypertensive compounds, but also their efficacy depending on consumption time. Although a plethora of studies has investigated food-derived compounds, such as phenolic compounds or peptides and their impact on BP, only a few addressed the relevance of time consumption. However, it is known that BP and its main regulatory mechanisms show a 24-h oscillation. Moreover, evidence shows that phenolic compounds can interact with clock genes, which regulate the biological rhythm followed by many physiological processes. Therefore, further research might be carried out to completely elucidate the interactions along the time–nutrition–hypertension axis within the framework of chrononutrition.

## 1. Introduction

Hypertension (HTN) is defined as a long-term condition associated with persistent high blood pressure (BP) levels. It is considered as a major cardiovascular disease (CVD) risk factor and, therefore, a global public health challenge. Remarkably, as a matter of fact, more than a half of the hypertensive population shows other CVD-related factors, such as obesity, being overweight, diabetes, metabolic syndrome, hyperlipidemia, etc. [1].

Due to this, preventive and treatment-focused approaches to lower BP and slow down HTN progression play a key role in the reduction of CVD risk by decreasing diastolic BP (DBP) and systolic BP (SBP) at least 5 and 10 mm Hg, respectively [2]. Many of the strategies involve the use of antihypertensive drugs but also natural bioactive compounds, especially when HTN is still moderate. These compounds can exert their antihypertensive activity through different pathways, including the renin–angiotensin–aldosterone system (RAAS), endothelial function, oxidative stress or inflammatory response, particularly acting as angiotensin-converting-enzyme (ACE) inhibitors or potent antioxidants [3,4,5,6]. Moreover, recent studies with probiotics have revealed other BP mechanisms via gut microbiota modulation [7,8], as hypertensive patients exhibit a gut microbiota dysbiosis [9].

Regarding the efficacy of the consumption of antihypertensive compounds, administration time is a crucial factor that must be considered together with the dosage, source and ‘matrix effects’ that might affect bioaccessibility and bioavailability of the active molecules. The relevance of the moment of the day when the antihypertensive compounds are administered is directly related to the influence of biological rhythms, not only in BP oscillations but also in bioactive metabolization. Previous clinical studies and meta-analyses have demonstrated the high variability within the BP-lowering effects of food bioactives, such as phenolic compounds, that were significantly effective in some trials [10,11,12] but did not reduce BP in others [13,14,15]. These controversial results have also been noticed for antihypertensive food peptides [16,17].

Once the main mechanisms of BP regulation by which natural bioactive compounds exert their BP-lowering effect have been reviewed, the aim of this review is to collect evidence about the efficacy of these natural antihypertensive molecules depending on administration time and highlighting the involvement of biological rhythms.

## 2. Blood Pressure and Its Main Regulation Mechanisms

BP is defined as the force exerted by circulating blood against the walls of the large arteries during heart contraction. It depends on the volume of blood ejected by the heart contraction into the vessels, the elasticity of the walls of the arteries and the rate of blood flow through the large vessels [18]. Two types of BP can be measured: SBP and DBP. The first one is the maximum value of BP and corresponds to the ventricular contraction, the systole. This depends on the cardiac output and elasticity of the large arteries, among other factors. Regarding the second type, DBP is the minimum value of arterial BP and corresponds to the cardiac relaxation and is an indicator of vascular resistance. Its value is dependent on blood flow speed [19].

BP is meticulously regulated, as an increase or decrease in its value can induce HTN and/or CVD. Too much fluid in the vessels results in an increase in the BP, whereas too little bloodflow causes its drop, with the negative consequences that this produces [20]. Many metabolic complexes and systems are involved in the regulation of BP, such as the total body fluid volume, vascular system structure, autonomic nervous system and vasoactive hormones [21]. In this sense, the neurohormonal system maintains the cardiovascular homeostasis, mainly through the sympathetic nervous system and the RAAS. When the BP suffers a sharp decrease in cardiopulmonary volume, it results in a proportional decrease in the firing of afferent nerves to the brain; in response, the brainstem reduces the vagal activity to the heart and increases the sympathetic activity to the heart and resistance vessels. In these conditions, the suprarenal increases its release of epinephrine (and the release of neuronal norepinephrine can also occur) which causes tachycardia; there is also an increase in stroke volume and vasoconstriction of peripheral vessels and renal arteries, which is the main trigger of RAAS overactivation [22,23].

### 2.1. Renin–angiotensin–aldosterone System

RAAS plays an important role in fluid homeostasis and cardiovascular function, including maintenance of BP. In fact, several components of this system are the target for different drugs aiming to treat several CVD, such as HTN. Thus, regulation of this system is crucial to prevent these diseases. The first evidence of the existence of this system was found by Tigerstedt and Bergman in 1898, who observed an increase in BP in healthy rabbits injected with rabbit renal homogenates. This fact indicated the presence of a pressor substance in the renal tissue, which was called renin, [24]. In 1934, Goldblatt et al. [25] developed a model of HTN in dogs by producing renal artery stenosis in one of the two kidneys (2K1C, a renin–angiotensin-system (RAS)-dependent model of HTN) and later, a model in which one kidney was eliminated and a stenosis was produced in the renal artery, resulting in the second model (1K1C; which is a volume-dependent HTN). A couple of years later, and using these animal models, two research groups headed by E. Braun Menéndez (Argentina) and I.H. Page (USA) independently identified a new vasoactive substance in plasma. They postulated that this vasoconstrictor was obtained from the enzymatic action of renin, which was the enzyme released into the venous circulation by the ischemic kidney. This peptide was called hypertensin and angiotonin, which were mixed to create a definitive and unique term, angiotensin (Ang). More details of the discovery of the RAS can be consulted in Milei et al., 2010 and Basso and Terragno, 2001 [26,27].

**Figure 1:**
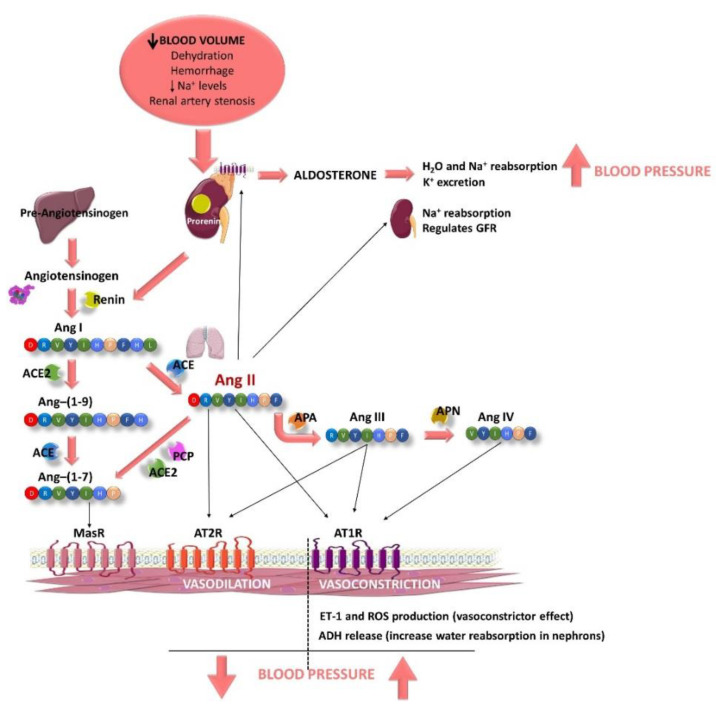
Schematic representation of the components of the renin–angiotensin–aldosterone system (RAAS) and some of their main effects, modulating blood pressure (BP). RAAS is activated when the blood volume decreases, plasma Na^+^ levels are low or a renal artery stenosis is suffered (2K1C experimental animal model). Juxtaglomerular cells (kidney) are activated to produce renin from prorenin, which is released to the bloodstream. Renin degrades hepatic angiotensinogen to form the angiotensin I (Ang I). Then, Ang I is hydrolyzed by the endothelial angiotensin-converting enzyme (ACE), mainly when it goes through the lung capillaries, releasing Ang II. Ang II produces vasoconstriction, acting directly on vascular smooth muscle cells after it binds to Ang type 1 receptor (AT1R). In addition, it also induces an increase in BP, stimulating (i) the production of reactive oxygen species (ROS) in the endothelium, (ii) the release of endothelin-1 (ET-1; an endothelial vasoconstrictor factor), (iii) the release of antidiuretic hormone (ADH) by the posterior pituitary gland, which produces reabsorption of water in the nephrons and (iv) the release of aldosterone by the suprarenal glands, which also produces reabsorption of water and Na^+^ and excretion of K^+^. Ang II also stimulates nephrons to Na^+^ reabsorption and regulates glomerular filtration rate (GFR). Ang II can also bind to AT2R, producing vasodilatation effects. Ang II is quickly degraded by aminopeptidase A (APA), releasing Ang III, which can bind to AT1R and AT2R producing the same effects described for Ang II. Ang III is further metabolized to Ang IV by the aminopeptidase N (APN), which also exerts central pressor effects via AT1R. Moreover, Ang II can also be hydrolyzed by ACE 2 or prolylcarboxypeptidase (PCP), producing Ang-(1-7). Ang-(1-7) can be also produced by the degradation of Ang-(1-9) by ACE. Ang-(1-9) is produced from Ang I after being hydrolyzed by ACE 2. Ang-(1-7) exerts nitric oxide (NO)–dependent vasodilatation via the G-protein–coupled Mas receptor (MasR).

This peptide/hormone system is activated by different causes, such as a decrease in blood volume due to dehydration, or hemorrhage or/and a decrease in Na^+^ levels (Figure 1). This fact produces the activation of the juxtaglomerular cells, located in the kidney afferent arterioles, producing the hydrolysis of prorenin, the precursor of renin [28]. Renin enters the bloodstream and reaches the liver, where this aspartyl protease triggers the cleavage of the angiotensinogen to form the decapeptide Ang I (Ang-(1-10)) [29]. This peptide is hydrolyzed by the ACE, mainly when it goes through the lung capillaries [30]. ACE is synthetized by the endothelial cells and its extracellular location in these cells helps the enzyme to interact easily with its substrate. As a result of its activity, the octapeptide Ang II (a potent vasoconstrictor) is formed [30]. In addition, ACE is also known to hydrolyze bradykinin, a vasodilator peptide, producing its inactivation and contributing to a reduction in the vasodilator factors [31]. It is worth noting that this enzyme is considered key in the BP regulation. Ang II, also known as Ang-(1-8), can bind to two different receptors: Ang type 1 receptor (AT1R) and Ang type 2 receptor (AT2R). These two receptors exert antagonist effects. The main actions of Ang II are associated with its binding to AT1R and are related to the development of CVD. This pathway is known as the ACE–Ang II–AT1R axis [32]. Ang II triggers intracrine, autocrine and paracrine responses with different physiological effects [28], as AT1R is located in most of organs and is coupled to different G proteins [33]. Ang II effects include producing vasoconstriction, acting directly on vascular smooth muscle cells [33]. It also increases total peripheral resistance through its vasoconstrictor effects on systemic arterioles [34]. This vasoconstrictor effect seems to be modulated by the endothelium, as it has been reported that Ang II can stimulate the release of different endothelial factors including the vasoconstrictor endothelin-1 (ET-1) [33] or reactive oxygen species (ROS) [35]. Moreover, Ang II stimulates the zona glomerulosa of the adrenal cortex to secrete aldosterone [28] and its release increases water and sodium reabsorption and potassium excretion in the distal tubule and collecting duct of the nephron [36]. It also activates the early proximal tubule (Na^+^–H^+^ antiporter) to Na^+^ reabsorption and regulates the glomerular filtration rate by the contraction of the efferent and afferent glomerular arterioles [37]. Furthermore, Ang II also acts in the hypothalamic level, firstly stimulating the sensation of thirst and consequently, promoting the intake of water. In addition, Ang II stimulates the release of the antidiuretic hormone (ADH, vasopressin) in response to the thirst in the posterior pituitary gland. This ADH acts on the collecting ducts of the nephron, increasing water reabsorption in this area, thus reducing urinary loss [28]. Altogether, these processes contribute to an increase in BP (Figure 1).

The half-life of Ang II in plasma is short (1–2 min) and it is degraded in its N-terminal position by aminopeptidase A, releasing another active peptide called Ang III (Ang-(2-8)) [33]. This peptide exerts agonistic effects to those shown by Ang II, including release of aldosterone, pressor and dipsogenic effects or stimulation of Na^+^ intake. It also binds to AT1R and AT2R to exert its effects [38,39,40]. Ang III is further metabolized to Ang IV (Ang-(3-8)) by the aminopeptidase N, which also exerts central pressor effects via AT1R, although Ang III can also bind to AT4R or insulin-regulated aminopeptidase (IRAP) [41]. Moreover, Ang II can also be hydrolyzed by other enzymes, including ACE 2 or prolylcarboxypeptidase, producing the peptide Ang-(1-7). Ang-(1-7) can be also produced by the degradation of Ang-(1-9) by ACE, previously obtained by the action of ACE 2 on Ang I [40]. This pathway is called the ACE2–Ang-(1-7)–Mas receptor (MasR) axis. It has been reported that Ang-(1-7) exerts nitric oxide (NO)–dependent vasodilatation, and antihypertensive, anti-inflammatory, antifibrotic and antiangiogenic effects via the G-protein–coupled MasR [42].

In addition to CVD, the role of RAS components in other diseases was recently revealed. For example, the role of ACE 2 in COVID-19 as SARS-CoV2 uses this enzyme to enter the mucosa and also modulates its gene expression [43]. ACE and Ang II also play a role in Alzheimer’s disease [44]: brain ACE expression was related to Alzheimer’s disease severity and amyloid-beta (Aβ) load and Ang II is responsible for the development of neurovascular damage and dysfunction via the AT1R pathway [45,46,47]. Moreover, agonists of brain AT2R and AT4R were suggested as potential drug candidates for the treatment of Alzheimer’s disease [44,48]. Thus, the RAS system continues to be of interest in the search for new treatments for different diseases.

### 2.2. Endothelial Function

**Figure 2:**
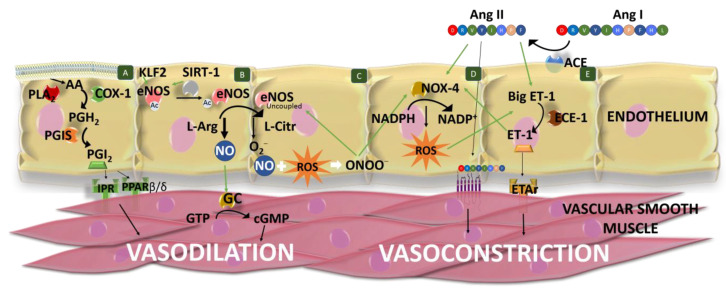
Schematic representation of the main vasodilator and vasoconstrictor factors produced by the endothelium. Cell (**A**) Phospholipase A_2_ (PLA_2_) releases arachidonic acid (AA) from membrane glycerophospholipids. AA is transformed into prostaglandin (PG) G_2_, which is further reduced to PGH_2_ by the cyclooxygenase 1 (COX-1). Finally, prostacyclin synthase (PGIS) converts PGH_2_ into PGI_2_, which exerts vasodilation of vascular smooth muscle binding to prostacyclin receptors (IPR) and peroxisome proliferator-activated receptor (PPAR) β/δ. (**B**) Nitric oxide (NO) is the main endothelial vasodilator factor which is synthesized through the oxidation of L-arginine (L-Arg) to L-citrulline (L-Citr) by the endothelial NO synthase (eNOS). *eNOS* expression is stimulated by Kruppel-like-factor 2 (KLF2), and eNOS is activated by sirtuin 1 (SIRT-1), which deacetylates it. Furthermore, SIRT-1 stimulates *eNos* transcription. NO diffuses into vascular smooth cells and produces vasodilatation by the activation of guanylate cyclase (GC), which converts GTP to cGMP. (**C**) eNOS can also produce ROS (superoxide anions) when it is uncoupled. These anions can scavenge NO, generating peroxynitrites (ONOO−), reducing NO bioavailability and NO-dependent vasodilatation. (**D**) ROS is produced by other enzymes, such as the NADPH oxidase 4 (NOX-4), which catalyzes the transfer of electrons from NADPH to molecular oxygen. NOX-4 activity is stimulated by angiotensin (Ang) II and peroxynitrite. (**E**) Ang II is formed from Ang I by the action of angiotensin-converting enzyme (ACE). Ang II produces the constriction of vascular smooth cells via Ang type 1 receptor (AT1R). Endothelin 1 (ET-1) is produced by the action of endothelin-converting enzyme 1 (ECE-1) on the big ET-1. ET-1 vasoconstrictor effects are mediated by its interaction with ETA receptors (ETAr), located in the vascular smooth cells. ET-1 synthesis or release is favored by Ang II and ROS. ET-1 can stimulate the vascular *Nox* expression. Green lines indicate stimulation/modulation.

In addition to RAAS, it is important to highlight the role of the endothelium in BP. The vascular endothelium is a tissue formed of a monolayer of endothelial cells, located between the bloodstream and the vascular smooth wall of the vessels [49]. It forms a highly selective impermeable barrier, which also secretes vasoactive compounds, in response to haemodynamic mechanical forces and hormones. These compounds act paracrinally to produce contraction and dilation of the vascular tissue, regulate vascular tone, vascular smooth muscle cell functionality, inflammation and immune response and maintain blood fluidity [50,51]. Figure 2 summarizes the main vasodilator and vasoconstrictor factors produced by the endothelium.

NO, initially called endothelium-derived relaxing factor, is the main endothelial vasodilator factor. It diffuses into vascular smooth cells, stimulating the conversion of guanosine triphosphate to cyclic guanosine monophosphate through the activation of the guanylate cyclase [52]. NO is also involved in angiogenesis, immune responses, inflammation (exerting anti-inflammatory effects in a normal healthy state) and inhibits white cell activation and platelet aggregation, among other effects [50,53,54]. In the endothelium, NO is synthesized through the oxidation of L-arginine to L-citrulline, in a reaction catalyzed by the constitutive isoform of the enzyme NO synthase (eNOS or NOS III), using as co-substrates nicotinamide-adenine-dinucleotide phosphate and oxygen [55]. This monomeric enzyme contains two domains (the reductase and oxygenase domains) that form dimers, which are considered the active form of the enzyme [54]. In the plasma cell membrane, this enzyme is found attached to caveolin-1, which acts by inhibiting the enzyme [56]. eNOS activation is produced in response to shear stress, vascular endothelial growth factor, HDL and intracellular Ca^2+^ levels [57]. It is a Ca^2+^-dependent activation, although eNOS can be also activated in its absence [58]. Moreover, eNOS activity depends on different cofactors (flavin adenine dinucleotide, flavin mononucleotide and (6*R*-)5,6,7,8-tetrahydrobiopterin (BH_4_)), the phosphorylation of different amino acids, post-translational lipid modifications [54,55] and the SIRT-1 activity, which deacetylates it. Furthermore, SIRT-1 stimulates *eNOS* transcription [59,60] and Kruppel-like-factor 2 (KLF2) stimulates *eNOS* expression [61].

Instead of NO, eNOS can also produce superoxide anions. This process is called “eNOS uncoupling”. It can happen when L-arginine or BH_4_ levels are low (BH_4_ stabilizes the eNOS dimer), or asymmetric dimethylarginine (an endogenous eNOS inhibitor) levels increase [62]. For example, reduction in BH_4_ levels can be produced by a decrease in BH_4_ production or by an increase in its oxidation due to excessive ROS levels, namely peroxynitrite [63,64]. Consequently, it generates a reduction in NO bioavailability and an increase in ROS levels, altering the endothelial function. This is associated with HTN and other CVD. Moreover, NO availability can also be reduced by superoxide anions, which can scavenge NO, generating peroxynitrites and avoiding NO-dependent vasodilatation [65]. Moreover, peroxynitrite can oxidize low-density lipoproteins which increase arginase activity, producing a reduction in L-arginine levels and also stimulating NADPH oxidases (NOX) and xanthine oxidase to produce ROS [62]; consequently, peroxynitrite and its ROS-induced production contribute to eNOS uncoupling. In addition to eNOS in its uncoupled state, endothelial cells produce ROS in the mitochondrial respiration and by means of xanthine oxidoreductase and NOX (mainly NOX-4 in these cells) [66,67]. Moreover, endothelial ROS production can be increased by different factors, such as Ang II action, as it can stimulate NOX-4 activity [68,69]. In the homeostatic state, the generated free radical is counter-balanced by endogenous antioxidant mechanisms, which can be enzymes, such as superoxide dismutase (SOD) or catalase (CAT), or non-enzymatic compounds, such as reduced glutathione (GSH) or ascorbate. An unbalance between ROS production and degradation results in oxidative stress, representing the main cause of endothelial dysfunction [51].

Prostaglandin (PG) or prostacyclin I_2_ (PGI_2_) is another important vasodilator factor produced by the endothelium, mainly in response to shear stress [49]. However, it is considered that it plays a secondary role in vasodilation, exerting its effect mainly when the levels of NO are not high enough [70]. This factor is synthesized by a multi-step enzyme-catalyzed reaction [71]. Firstly, phospholipase A_2_ releases arachidonic acid from membrane glycerophospholipids, whose activation depends on Ca^2+^ levels [72]. Secondly, the free arachidonic acid is transformed in PGG_2_, which is further reduced to PGH_2_ by the action of the cyclooxygenase (COX). This enzyme shows oxygenase and peroxidase activities [71] and the predominant isoform in endothelial cells is COX-1 [73]. Finally, prostacyclin synthase (PGIS) converts PGH_2_ into PGI_2_. The effects of PGI_2_ are mediated by its binding to cell surface prostacyclin receptors (IPR) and intracellular peroxisome proliferator-activated receptor (PPAR) β/δ. Activation of both pathways produces a multi-step reaction that results in a reduction in intracellular Ca^2+^ levels of vascular smooth cells and a further vasodilation of the vessel [71]. Moreover, it is known that NO induces the release of PGI_2_ and vice versa [49]. PGI_2_ can also act on juxtaglomerular apparatus, inducing the release of renin by kidney [74].

On the other hand, the endothelium also synthesizes and releases vasoconstrictor compounds, such as ET-1 which is also involved in vascular and myocardial hypertrophy and promotes inflammation as it stimulates the release of interleukins (IL-6, IL-1 and IL-8) [75]. ET-1 is produced in different steps, comprising the hydrolysis of prepro-ET-1 into big ET-1 by proteases and the further hydrolysis in Trp-21 of big ET-1 in its active form ET-1, catalyzed by the endothelin-converting enzyme 1 (ECE-1) [76]. This process is tightly regulated by different factors. In this regard, ET-1 synthesis or release is favored by Ang II, ADH, ROS, cytokines (tumor necrosis factor-alpha and IL-1), norepinephrine, thrombin or shear stress, while it is reduced by NO, atrial natriuretic peptide, cyclic nucleotides and KLF2 [77,78,79]. The vasoconstrictor effects of ET-1 are mediated by its interaction with ETA and ETB receptors (mainly ETA receptors), located in the vascular smooth cells [80]. However, ET-1 can also bind to ETB receptors in the endothelial cells, presenting an opposite effect to that showed by ETA activation. Specifically, ET-1 via ETB favors the release of endothelial prostacyclin and NO, ET-1 clearance, and inhibits ECE-1 expression [79]. In addition, it has been observed that ET-1 can stimulate the vascular *Nox* expression [81]. Another vasoconstrictor produced by the endothelium is Ang II, as ACE is expressed in endothelial cells. This local Ang II helps to maintain normal BP, although it is not essential [31,82]

The balanced release of vasoconstrictor and vasodilator factors by the endothelium leads to a controlled homeostasis of vascular tone and BP [49]. The imbalance between vasodilator and vasoconstrictor factors may trigger the development of some CVD, such as HTN.

## 3. Biological Rhythms and Blood Pressure

Many physiological processes including BP and heart rate follow a biological rhythm. These rhythms are organized in cycles that allow the organisms to adapt to constant changes in their environment, such as light and dark periods or even seasonal changes, thus optimizing their metabolic functions and energy expenditure [83]. In mammals, these rhythms are controlled by synchronized endogenous clocks, which are located both in the central nervous system and in peripheral areas throughout the body. Because of this synchronization and their connection to the environment [83,84], these clocks are able to modulate many biological processes, such as neuronal, endocrine, metabolic and behavioral functions [85]. The main factor that regulates and controls the endogenous clocks is the 24-h light/dark cycle of Earth, also called the photoperiod. Nevertheless, other environmental or behavioral factors, such as meal timing and exercise are also essential in the modulation of these clocks [86].

### 3.1. Molecular Machinery behind Circadian Rhtyhms

In mammals, the central clock of the circadian rhythms, which synchronizes all existing peripheral clocks, is located in the suprachiasmatic nucleus (SCN), specifically in the ventral periventricular zone of the anterior hypothalamus. The SCN receives information about external light through its connection with the retina and sends it to other organs, thereby generating behavioral and biological rhythms [87].

**Figure 3:**
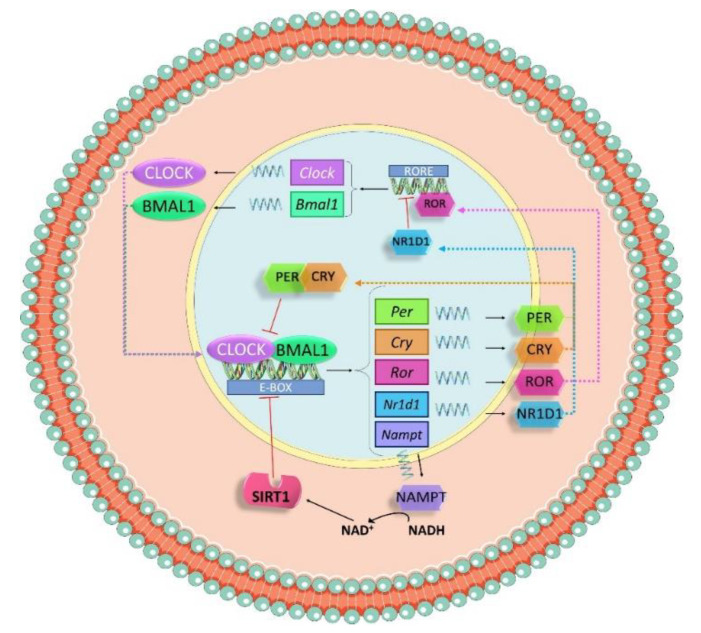
Schematic representation of the molecular mechanism of the molecular clock. Circadian locomotor output cycles kaput (CLOCK) and brain and muscle Arnt-like 1 (BMAL1) dimerize to bind to the E-box elements in promoter regions of clock-controlled genes such as *Per* and *Cry*. CRY and PER form a complex that represses the heterodimer CLOCK–BMAL1 in the nucleus, inhibiting the transcription of clock genes by a negative feedback loop within a 24-h period. The heterodimer CLOCK–BMAL1 also drives a regular expression of nicotinamide phosphoribosyltransferase (*Nampt*). NAMPT triggers the release of NAD^+^, a cofactor needed for Sirtuin 1(SIRT1), which modulates the activation of clock genes via deacetylation of histones.

At the molecular level, the circadian clock is controlled by a set of genes called clock genes, which codify many transcription factors that undergo an autoregulatory transcription–translation feedback loop (Figure 3). The most important clock proteins are circadian locomotor output cycles kaput (CLOCK) and brain and muscle Arnt-like 1 (BMAL1). They dimerize to bind to the E-box elements in promoter regions of clock-controlled genes, such as *Per1*, *Per2* and *Per3* (period 1, 2 and 3) and *Cry1* and *Cry2* (cryptochrome 1 and 2). CRY and PER form a complex that represses the heterodimer CLOCK–BMAL1 in the nucleus, thus inhibiting the transcription of clock genes by a negative feedback loop within a 24-h period [88].

Additionally, the system is also modulated by a secondary loop in which *Bmal1* expression is controlled by nuclear receptors, RAR-related orphan receptor alpha (RORα) and nuclear receptor subfamily 1 group 1 member 1 (NR1D1). Both proteins are downstream products of the CLOCK–BMAL1 pathway and can bind with the ROR/REV-ERB-response element (RORE) in the promoter sequence of *Bmal1*. RORα acts as an activator of the transcription, while NR1D1 is an inhibitor [89].

Not only do transcriptional and translational loops control the activation and repression of clock genes, but also post-translational modifications. The heterodimer CLOCK–BMAL1 drives a regular expression of nicotinamide phosphoribosyltransferase (*Nampt*) and NAMPT triggers the release of NAD^+^, a cofactor needed for the NAD^+^-dependent deacetylase sirtuin 1(SIRT-1), that modulates the activation of clock genes via deacetylation of histones [90].

### 3.2. Circadian Blood Pressure Patterns

**Figure 4:**
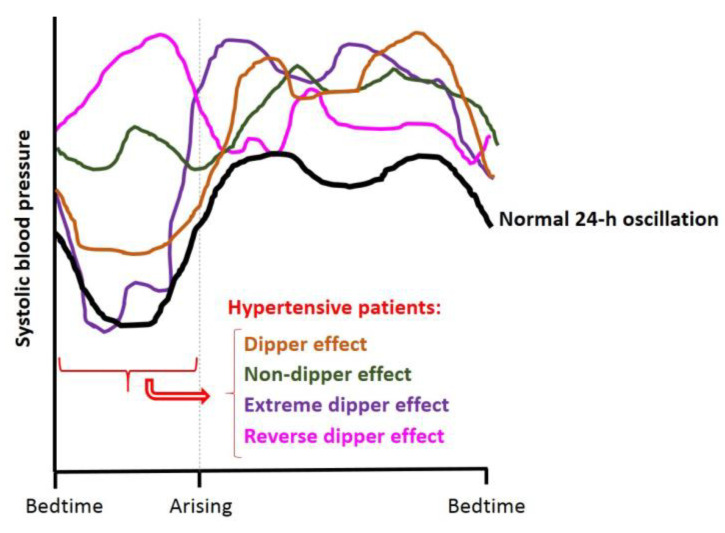
Schematic representation of a normal dipper 24-h BP cycle (black line) and different BP behavior in hypertensive patients: dipper (orange line), non-dipper (green line), extreme dipper (violet line) and reverse dipper/riser (pink line) effect. Adapted from Hermida et al., 2007 [95].

It is well known that BP in mammals follows a 24-h rhythm. Like heart rate, BP in humans reaches its highest value after awakening [91,92], where higher prevalence of myocardial infarction, sudden cardiac death and myocardial ischemia is observed. Moreover, it also shows a second peak in the afternoon (~7:00 p.m.). Conversely, during sleep, there is a drop (~10–20% compared with daytime values) in BP, known as the dipper effect [92,93,94]. This effect is used to classify subjects as “extreme-dippers” (SBP is reduced ≥ 20% when compared with the daytime BP value); “dippers” (SBP is reduced between 10–20% when compared with their daytime BP); “non-dippers” (SBP does not show a drop, or it is less than 10%) and “inverse-dippers or risers” (SBP increases on nighttime BP value, instead of showing a drop) [95]. Figure 4 shows a representation of all these BP circadian patterns. Dippers are associated with cardiovascular health, while “non-dippers” present a higher cardiovascular risk and correlate with higher end organ damage in different tissues [96]. Thus, these circadian BP fluctuations are used as predictors for CVD [97]. Moreover, it has been seen that hypertensive patients can follow different cycle patterns, which are classified as dipper, non-dipper, extreme dipper and reverse dipper depending on BP behavior during the night [95].

BP regulation mechanisms are under the control of the biological clock machinery [92,95,98,99]. In fact, biological peripheral clocks are found in the heart, blood vessels and vascular endothelial cells [100]. Experimental studies in murine models have proven that BP and heart rate follow a 24-h oscillation, which is disrupted by abolishing the SCN and thus the central clock functions [95]. Interestingly, in some transgenic hypertensive rats, such as the TGR(mRen2)27 rats, the biological rhythmicity of BP is lost, showing an increase during the rest phase, contrary to the normotensive fluctuations [94]. Specifically, several studies showed that *Bmal1* knock-out (KO) male mice exhibit a decrease in BP values when compared with normotensive mice [101,102,103] and a loss of the circadian BP variations in these animals [102,104]. Another study revealed that the effects of *Bmal1* on BP is dependent on organ. Thus, the deletion of the Bmal1 gene in smooth muscle produced a BP decrease and modified BP rhythm, while the deletion of *Bmal1* in cardiomyocytes did not produce the same effects [105]. In addition, *Bmal1* KO mice had endothelial dysfunction [106]. Moreover, studies carried out in kidney-specific cadherin *Bmal1* KO mice under a K^+^-restricted diet revealed that BMAL1 is involved in Na^+^ reabsorption in the distal nephron and collecting duct of nephrons, contributing to BP regulation [103]. Studies with *Clock* KO mice showed a hypotensive phenotype and a higher urine excretion of sodium, thus suggesting that this gene could be implicated in mechanisms related to the sodium transport in the nephron [107]. No changes in BP rhythms were observed in these animals. Moreover, Per1 KO mice showed reduced BP and higher levels of ET-1 in the kidney in comparison with Wild-Type (WT) controls, due to the role of *Per1* in the renal sodium reabsorption and excretion [108]. Male *Per1* KO mice were also more sensitive to a high salt diet plus mineralocorticoid treatment, which produced an increase in the mean arterial pressure and a non-dipper phenotype [109]. However, this non-dipper effect produced by the treatment was not observed in female *Per1* KO mice [110]. In addition, *Per2* KO mice showed endothelial dysfunction and a slight reduction in diastolic BP [111,112]. Finally, *Cry1/2* KO mice showed salt-sensitive HTN associated with high synthesis of aldosterone [99,113].

The relation between BP and biological rhythms is mechanistically explained by sympathetic activation during the day, which triggers a release of epinephrine and norepinephrine that is especially high during morning hours [97]. This leads to the activation of the RAAS system and its components that also have their maximum concentration peak just before awakening [92,93]. In this regard, Kawasaki et al. (1990) evidenced in healthy subjects that the 24-h pattern of the concentration of total renin, active renin and aldosterone plasma and renin activity follows a circadian rhythm. In addition, the maximum concentrations of these parameters were observed at early morning (~05:45–09:00 h) except for total renin concentration that was found at early afternoon (14:42 h) [114]. Moreover, Richards et al. observed a weak correlation between circulating levels of renin and Ang II in plasma and BP in healthy subjects [115]. In addition, a reduction in the endothelial vasodilator function and a decrease in parasympathetic activity explain the maximum value of BP at the beginning of the day [92]. Regarding the nighttime pattern of BP, it can be explained by parasympathetic activity, which rises and exerts the opposite effect to sympathetic activity. Thus, RAAS activation decreases and, consequently, Ang II, ACE and aldosterone concentrations in blood are reduced [93]. Furthermore, NO concentrations and endothelial function increase. All these events boost the dipper effect seen during the asleep phase [92,93,94].

It is known that circadian rhythms also regulate endothelial NO production, since total expression of eNOS is under control of the circadian clock and peaks during the active-period [116,117]. Although the NO produced by eNOS is key in BP regulation, it seems that eNOS is not involved in the circadian rhythmicity of BP [118,119]. Although experiments carried out in *eNos*^−/−^ mice and WT mice administered L-Nω-nitro arginine methyl ester (L-NAME, a non-specific NOS inhibitor) showed a BP increase in respect of WT mice, they maintained the 24-h BP rhythmicity [118]. Additionally, the gene expression of GTP cyclohydrolase-1 and dihydrofolate reductase, enzymes involved in the synthesis of the eNOS cofactor BH_4_, also have a circadian rhythm in WT mice, reaching a peak during the active period simultaneously to eNOS [106,120]. This rhythmicity was directly regulated by the circadian clock, as it was lost in the aorta of *Bmal1* KO mice. These animals also exhibited higher levels of superoxide than those shown by WT mice, which was associated to the eNOS “uncoupling” [106]. Moreover, circadian rhythms of *Nox-4* gene expression were also observed in WT murine heart and human aortic endothelial cells which were under BMAL1 control [121]. Furthermore, it has been observed that plasma ET-1 (vasoconstrictor) levels, and urinary ET-1 levels follow a 24-h cycle in humans [122,123]. Specifically, plasma ET-1 levels were shown to be higher in the morning compared with the afternoon. Moreover, it was observed that the ET-1 excretion rhythm corresponds with the rhythm followed by sodium excretion and the latter, in turn, showed a similar circadian cycle to BP [122]. Moreover, expression of ET-1 (Edn1) and ET-1 receptors (Ednra and Ednrb) genes also exhibited a 24-h rhythm in C57BL/6J mice. Specifically, Edn1 expression levels showed the acrophase in the dark phase in all tissues, while it was tissue-dependent in the case of the Ednra and Ednrb expression levels [123].

## 4. Hypertension

HTN is a chronic condition that causes a persistent elevation of BP in the vessels to at least 90 mm Hg in DBP and 140 mm Hg in SBP [124]. According to the World Health Organization, over than a billion people suffer from HTN around the world and, most of them, do not have awareness of it so, in most cases, HTN is not controlled and constitutes a major and relevant risk factor for CVD, which is still the leading cause of mortality worldwide [125]. In European countries, the prevalence of HTN is around 30–45% and the percentage increases with age. Although men and women have the same risk, HTN usually appears in men at an earlier age and this potential risk is increased in women after menopause [126].

According to the European Society of Hypertension and the European Society of Cardiology, HTN can be classified as grade 1 (DBP 90–99; SBP 140–159 mmHg), grade 2 (DBP 100–109; SBP 160–179 mm Hg) and grade 3 (DBP ≥ 110; SBP ≥ 180 mmHg), as well as primary (also essential or idiopathic) or secondary HTN [124]. Moreover, there is another condition called prehypertension, which includes people in process of HTN development (DBP 80–99; SBP 120–139 mmHg). Primary HTN, the most extended (≈95% of HTN) emerges due to unknown causes but some factors are strongly correlated such as age, diet or pharmacology treatments [127,128]. Moreover, genetics and environmental factors such as obesity, sedentary lifestyle, alcohol, high salt/Na^+^ diet, K^+^/vitamin D deficiency, etc. can also be involved and lead to an earlier appearance of HTN and CVD [128,129]. When HTN is caused by other pathologies (renal, thyroid, hormonal, vascular or metabolic disorders), it is classified as secondary HTN [127].

The high BP levels recorded in hypertensive patients originate in the disruption and functional alteration of regulation systems, such as RAAS and other disorders, for example, endothelial dysfunction which starts with an imbalanced secretion of vasodilator and vasoconstrictor molecules. When the endothelium is damaged, pro-inflammatory and pro-thrombotic factors (chemokines, cytokines, and adhesion molecules) are released and interact with leucocytes and platelets, thereby provoking the loss of integrity of endothelial cells that can be detached from the vascular wall. In addition, the increase in pro-inflammatory chemokines contributes to T cells and macrophage infiltration, generating tissue injury [130].

Another consequence caused by HTN is ROS overproduction. In this situation, ROS content might reach levels that cannot be buffered by endogenous antioxidant mechanisms, leading to oxidative stress. As was previously mentioned in Section 2, “uncoupled” eNOS and NOX are particularly relevant in endothelial ROS generation [49,62]. Since the RAAS is overactivated in HTN, Ang II is overproduced, affecting BP via increasing ET-1 and ROS production (stimulating NOX-4 activity and eNOS uncoupling) (Figure 2) [68,79]. Moreover, ROS overproduction triggers adipose inflammation, glucose intolerance and insulin resistance. In addition, the generated superoxide anions can react with NO, increasing peroxynitrite production and decreasing NO bioavailability. In turn, peroxynitrite is linked to alterations of redox-sensitive genes and transcription factors [49,131]. The results of all these processes negatively affect the vascular physiology, contributing to damage progression related to CVD [131].

Moreover, biological rhythm disruptions can lead to HTN or CVD clinical pictures. For instance, populations such as shift workers (particularly nighttime shift workers) that suffer a misalignment in the natural light–dark schedule showed alterations in their BP levels in comparison with daily workers. In addition, alterations in sleep duration were demonstrated to be associated with cardiometabolic disorders development [132,133,134]. Furthermore, as was mentioned in Section 3.2, the deletion of *Per1* in male mice in combination with the consumption of a high salt diet plus mineralocorticoid treatment produced an increase in the mean arterial pressure and a non-dipper phenotype, which is associated to HTN [109].

### 4.1. Treatments for Hypertension

Since HTN is assumed as a relevant health problem worldwide, a plethora of potential preventive and corrective solutions have been investigated. Among them, well-established and novel pharmacological approaches have been defined and are efficient to manage HTN in most of the cases; however, they can exert side effects in some patients and they are not suitable for prehypertensive patients. Thus, new bioactive compounds obtained from natural sources or food have emerged as an adequate alternative. Moreover, lifestyle measures such as salt/alcohol intake restrictions, exercise, diet, weight loss, etc. must not be forgotten [135,136,137].

#### 4.1.1. Pharmacological Treatments

The therapy against HTN includes a wide range of drugs that interact and inhibit RAAS components and other HTN agonists. Thus, one of the most investigated and prescribed treatments are those that involve ACE inhibitors, such as captopril or lisinopril, as well as benazepril, ramipril and imidapril. While ACE inhibitors prevent the production of Ang II, different Ang II receptor blockers (ARB), such as valsartan and olmesartan, reduce its action, thereby avoiding blood vessel constriction [138,139]. Furthermore, inhibitors of β-adrenergic receptor blockers are utilized to attenuate heart β-adrenoreceptor activation. These compounds, such as propranolol, are commonly used combined with other drugs and their proposed mechanism of action includes heart rate and cardiac output reduction, renin release inhibition, venous return and plasma volume reduction and vascular compliance improvement, among others [140]. Furthermore, during recent decades, calcium channel blockers (amlodipine, felodipine, isradipine) have been widely used (monotherapy or combined therapy) because of their good tolerability by hypertensive patients and their effectiveness in reducing BP via blocking calcium entry into cardiovascular cells and thus triggering a vasodilator effect [141]. Moreover, different diuretic drugs are used in several cases of HTN, acting in several areas of nephrons. For instance, thiazide-type diuretics block Na^+^-Cl^-^ cotransporters in the distal convoluted tubule and, consequently, reduce Na^+^ reabsorption. Also, loop-active agents, such as torasemide, block Na^+^-K^+^-Cl^-^ cotransporters in the thick ascending limb of the loop of Henle and potassium-sparing diuretics act on Na^+^-K^+^ pumps, decreasing K^+^ excretion in the late distal tubule and collecting duct [142].

Despite the high diversity of pharmacological treatments than can be prescribed for the different HTN grades, a substantial percentage of hypertensive patients show uncontrolled BP because of intolerance or nonadherence to the abovementioned antihypertensive agents [135,143]. Thus, novel drugs, devices and procedures are being investigated in preclinical and clinical studies. In this sense, several drugs are in preclinical or phase-I of development, such as those based on the inhibition of dopamine β-hydroxylase, aminopeptidase A and Na^+^/H^+^ exchanger 3, as well as vaccines targeting Ang II and AT1R and antioxidants such as vitamin D. Other agents are at a more advanced stage (phase-II/III), such as newer mineralocorticoid receptor antagonists, inhibitors of aldosterone synthase, vasopeptidases and soluble epoxyde hydrolase, and agonists of natriuretic peptide A and vasoactive intestinal peptide receptor 2 [135,143].

#### 4.1.2. Natural Bioactive Compounds

Although antihypertensive drugs have shown effectiveness against HTN, other alternatives are considered when the disorder is in development. For instance, diet modifications combined with healthy lifestyles were shown to prevent and alleviate the disruptions caused by the disease [137,144]. Besides recommended eating plans, such as DASH (Dietary Approaches to Stop HTN), that advises a reduced salt/sodium and saturated fat intake and includes fruit and vegetables, specific natural matrices have been subjected to investigation to obtain bioactive compound-enriched products [144,145]. Thus, bioactive compounds extracted from natural sources and food have been targeted to develop novel approaches that might have a place in a patient’s diet. Among these compounds, several molecules including peptides, phenolic compounds, vitamins, carotenoids, alkaloids and organosulfur compounds have shown antihypertensive potential, although not all the mechanisms of action are fully elucidated [137,146].

These antihypertensive compounds are searched and selected, based on their ACE inhibitory (ACEi) properties. This is due to the fact that pharmacological ACE inhibitors are the first-line treatments for HTN and an inhibition of ACE results in an effective BP reduction [147]. In this sense, protein hydrolysates and peptides obtained from varied food matrices exert significant ability to inhibit or reduce ACE activity. For instance, those prepared from vegetal products and by-products, such as from fruits, legumes, cereals, wine lees, pseudocereals, herbs and spices, etc. showed potent *in vitro* ACEi activity and, in some cases, *in vivo* studies were conducted to validate the BP-lowering effects [148,149,150,151]. Moreover, animal sources, such as dairy products, meat, fish or eggs and also specific by-products from these industries, such as chicken feet, led to potent ACE inhibitors, as well as other alternative matrices, such as algae or mushrooms [149,152,153,154,155,156,157,158]. In addition, it has been reported that some food matrices including different dairy products, such as kefir or cheese and unfiltered olive oil, can also contain ACE inhibitory and /or antihypertensive peptides [8,159,160,161]. Peptide length and amino acid position in the chain is very important to inhibit ACE. Thus, peptides of small size (3–12 amino acids), the presence of hydrophobic amino acids or those containing hydrophobic-branched side chains at the C-terminal end and the presence of the branched aliphatic amino acids at N-terminal position were related to a higher ACEi activity than peptides not presenting these characteristics [157].

Other important compounds with potent ACEi activities are the phenolic compounds. Their activity has been linked to the number of the hydroxyl group on the benzene ring [162]. Moreover, the ACEi activity of the flavonoid family has been associated with the presence of the catechol group in the B-ring and the ketone group at the C4 and the double bond between the C2 and C3 positions in the C-ring of the structure [163]. In addition to individual phenolic compounds, ACEi activities were reported for phenolic-rich extracts or beverages obtained from wine, tea, legumes, barley, winery by-products and algaes among others [3,164,165,166,167].

In addition to ACEi activity, protein hydrolysates were found in macroalga Palmaria palmate [168], lima bean [169], soy bean [170] and flaxseed [171], phenolic-rich extracts from tree peony petals [172] and from leaves of *Cuphea ignea* A. DC. [173] with *in vitro* renin-inhibitory activity, some of them with a BP-lowering effect *in vivo*.

Some of these ACE or renin-inhibitory compounds are able to modulate the RAAS components after being consumed by hypertensive animals. For instance, such is the case of *Hibiscus sabdariffa* L. extracts, which produced a reduction in plasma Ang II, ACE and aldosterone levels in L-NAME–induced hypertensive rats [174]. However, although natural antihypertensive compounds are mainly searched and selected based on their activity on RAAS, it is very common that they can decrease BP by acting on other BP regulation pathways after ingestion by hypertensive animals. For instance, they can act on the endothelium, restoring the endothelial dysfunction and oxidative stress associated with HTN. Thus, they can increase the NO bioavailability in animals treated with bioactive compounds by favoring the NO release (acting on the activity and expression of eNOS and SIRT-1), as well as decreasing ROS levels (downregulating *Nox* expression and increasing the activity or upregulating the expression of different endogenous antioxidant enzymes including SOD, CAT, gluthatione peroxidase, heme oxygenase, gamma-glutamylcysteine synthetase and glutathione S-transferase enzymes) [3]. A decrease in plasma levels in the vasoconstrictor ET-1 and a downregulation of its gene expression (*Edn1*) [3] have also been reported. For instance, a chicken foot hydrolysate, selected according its good ACEi activity, showed a potent antihypertensive effect after its acute administration to spontaneously hypertensive rats (SHR) and after its long-term administration to diet-induced hypertensive rats (CHR) [156,175]. It was found that this bioactive hydrolysate upregulated aortic *Sirt1* expression, downregulated *Edn* expression and increased hepatic GSH levels (the main endogenous antioxidant) after its long-term administration to CHR [175]. In addition, the peptide sequence AVFQHNCQE was found in the hydrolysate, showing good *in vitro* ACEi activity (IC_50_ = 44.8 µM) and potent antihypertensive effects in SHR [157]. Moreover, administration of an acute dose (10 mg/kg body weight) of this chicken foot-derived peptide to SHR produced an improvement of endothelial function and oxidative stress in these animals, as a downregulation of aorta *Edn1* and *Nox-4* gene expression was found and an increase in hepatic GSH levels in respect of control SHR [176]. Moreover, the peptide AVPYPQ, identified in a kefir beverage, showed *in vitro* ACEi, antioxidant and free radical scavenger activities [177,178,179]. Administration of this peptide (10 mg/kg/day) for 7 days to 2K1C mice, an animal model of secondary HTN, reduced both BP and heart rate. It produced a ROS-level reduction in vascular smooth muscle cells (acting on ROS production pathways: NOX and mitochondria), attenuated aortic thickening and reduced structural damage in the aortic endothelium in comparison with control 2K1C mice [179].

Similarly to these abovementioned peptide examples, the mechanisms involved in the BP-lowering effect of a liquid fraction of wine lees (rich in phenolic compounds) in SHR, selected also by its ACEi effect, was an improvement of endothelial function (downregulating of *Nox-4* and *Edn* gene expression and upregulating of *eNos* and *Sirt1* gene expression) and of oxidative stress (increasing hepatic GSH levels) [147,180,181]. Another example is grape seed proanthocyanidin extract (GSPE), that evidenced ACEi and antihypertensive effects in several hypertensive animal models (SHR and CHR) and produced an upregulation of *Sirt1* expression and a downregulation of *Edn* expression in the aorta of CHR administered with the extract (25 mg/kg) for 3 weeks [182]. Additionally, it decreased plasma ET-1 levels, upregulated aorta *eNos* expression and downregulated aorta *Nox* expression in CHR consuming a single dose (375 mg/kg) of the extract [183]. Moreover, NOX-activity modulation has been reported for individual phenolic compounds, such as catechins, hesperidin and curcumin that can be obtained from tea, citrus fruits and turmeric, respectively [184].

It is worth noting the *in vitro* antioxidant effects shown by some of these natural compounds, such as phenolic compounds. Thus, these compounds can scavenge or attenuate the generation of ROS within the oxidative stress status linked to HTN and, therefore, hinder the disease progression. A great number of works have been carried out referring to the antioxidant activity of phenolic compounds. This antioxidant and antihypertensive potential was observed for phenol-rich products from fruit and vegetables, such as grapes, cherries, berries, tea leaves, etc. [185,186,187].

In addition to dietary bioactive compounds, the importance of probiotics in the BP regulation was reported. For example, the administration of the probiotic kefir beverage (0.3 mL/100 g bw) to SHR for 60 days produced a significant reduction in BP and heart rate in these animals [188,189]. Its antihypertensive effect was associated with an improvement of endothelial dysfunction. Specifically, it improved the responsiveness of vessels (aorta) to acetylcholine-induced endothelium-dependent vasorelaxation in response to reduced levels of aortic ROS (•O_2_^−^, H_2_O_2_, and ONOO^−^/•OH^−^), increased levels of NO in aorta and increased the circulating endothelial progenitor cells levels, in respect of control SHR [189]. The correlation between probiotics administration and endothelial dysfunction amelioration was also noticed after *Lactobacillus fermentum* or *Bifidobacterium breve* supplementation (1 billion colony forming units per day) to SHR for 18 weeks [190]. Furthermore, administration of the probiotic VSL#3 (50 billion bacteria/kg/bw/daily) to chronic bile-duct-ligated (CBDL) rats prevented endothelial dysfunction, oxidative stress, inflammation and overactivation of aorta RAS associated with this animal model [191]. Another example is the administration of *Lactobacillus coryniformis* CECT5711 to high-fat-induced obese mice for 12 weeks, which improved the endothelial dysfunction and restored ROS levels (reducing NOX activity and increasing antioxidant enzymes) of these animals in respect of control mice [192].

## 5. Antihypertensive Compounds and their Role in Biological Rhythms

### 5.1. Pharmacological Treatments and Circadian Rhythms

Traditionally, the clinical approaches for the management of HTN is based on the modulation of the treatment dose or the combination of different drugs, ignoring how the rhythmicity of the pathology impacts on the efficacy, kinetics, dynamics and toxicity of the therapeutic products. In this regard, the study of chronopharmacology in antihypertensive treatment aims to synchronize drug concentrations with the circadian rhythms of the disease, in order to increase the effectiveness and to reduce the side effects of the treatment [193].

Patients with HTN generally take BP-lowering medication in the morning [194], despite several clinical trials having demonstrated that the efficacy of the available antihypertensive drugs varies depending on whether they are ingested in the morning or evening [195]. For example, ACE inhibitors, i.e., drugs such as captopril, ramipril or lisinopril, generally improved tolerance and antihypertensive efficacy when they were taken at bedtime [196]. By contrast, treatments with other drugs, such as imidapril, did not exert different effects between morning and evening dosage or, in the case of benazepril, even resulted in a better antihypertensive activity after morning administration [197].

In a similar manner to ACE inhibitors, treatments with ARB in monotherapy enhanced the reduction in BP values when dosed at a rest phase [196]. Evening treatments with valsartan, both in dipper and non-dipper volunteers, showed a reduction in asleep BP values and normalized the 24-h BP pattern [93]. Similarly, olmesartan consumed at bedtime was more effective in reducing SBP/DBP, as well as increasing the BP decline produced during sleep-time in essential hypertensive patients than the same drug consumed in a morning dosage [198].

Concerning other therapies, calcium channel blockers showed a reduction in BP independently of the treatment-time regimen [199]. However, the diuretic torasemide was much more effective when administered at bedtime compared with morning time [200]. Little information is available regarding the relationship between dosing time and efficacy of β-adrenergic receptor blockers. Only nebivolol, dosed at bedtime, maintained its efficacy throughout the daytime and slightly attenuated the nocturnal reduction in antihypertensive effect generally observed in β-adrenergic receptor blockers [201].

### 5.2. Bioactive Compounds and Circadian Rhythms

As mentioned above, alternative therapeutic approaches are considered for preventing the development of HTN. Considering that drugs used to treat diseases such as HTN exert differential effects depending on administration time, as well as bioactive compounds targeting the same molecular pathways than drugs, it is logical to think that the effectiveness of bioactive compounds could also vary depending on administration time. Thus, chrononutrition, a discipline that studies the relationship between temporal eating patterns, biological rhythms and metabolic health, has emerged as a potential therapeutic option [85]. Specifically, an appropriate composition of the diet and the timing of food intake can preserve the circadian rhythmicity and promote healthy metabolic and cardiovascular systems [202]. In this regard, phenolic compounds have been described as compounds able to regulate BP and endothelial function, and which can also interact with the circadian rhythm by affecting the expression clock genes (see [88,203] for more details). Consequently, as well as for conventional drugs, the dosing schedule of phenolic compounds could modify their ability to restore metabolic disorders [203].

One experimental study in rats revealed that resveratrol, a well-known antioxidant, generated different oxidative effects depending on the administration schedule [204]. Specifically, the study analyzed the levels in heart, liver and kidney of thiobarbituric acid reactive species (TBARS), lipid peroxidation-derived products that have been correlated with cardiovascular risk factors such as HTN [205]. Measurements showed a high decrease in TBARS values after light span administration and, conversely, a dramatic increase in TBARS concentrations when resveratrol was administered during the dark phase. Based on this evidence, the authors concluded that time of consumption of resveratrol may impact its function in the cardiovascular system, thus recommending morning intake in humans to obtain better therapeutic results.

Similarly, GSPE has widely shown potential beneficial effects on different metabolic syndrome components, including antihypertensive properties in an obese rat model [182]. In another study with rats on standard diet, GSPE also modulated the circadian rhythm by targeting BMAL1 acetylation in the liver, only after administration at the beginning of the dark (active) phase [206]. Consequently, this phenolic extract modulated the levels of NAMPT and NAD^+^ in the liver depending on the time of GSPE administration, thus integrating the impact of GSPE on both biological rhythms and metabolic pathways [206].

There are several studies in rodents, as well in humans, highlighting the antihypertensive effects of epigallocatechin-3-gallate (EGCG), a phenolic compound found mostly in green tea [207,208]. Interestingly, in a murine model of diet-induced obesity, only during the dark phase, did this compound restore the alteration of the biological rhythms due to the diet by regulating the expression of *Bmal1*, *Clock* and *Cry1* through the modulation of the SIRT1–PGC-1α loop, in liver and adipose tissue [209].

Moreover, it has been suggested that cacao liquor procyanidin (CLPr), an antihypertensive extract rich in phenolic compounds (epicatechins catechins and procyanidins) [88] could regulate clock gene expression in the liver by modulating the secretion of glucagon-like peptide-1 in mice [210]. Additionally, CLPr administration, only at the light period (rest phase), suppressed postprandial hyperglycemia by activation of the AMP-activated protein kinase (AMPK) and the resulting translocation of glucose transporter type 4 (GLUT4), thus demonstrating that the beneficial effects of this compound on metabolic syndrome was related to its administration timing [211].

It is undeniable that circadian oscillations should be considered in the traditional and alternative therapies for HTN. Unfortunately, despite the increasing evidence of the importance of chrononutrition in the management of metabolic disorders, and the evidence describing the cardioprotective properties and the modulatory effect of phenolic compounds over the regulation of clock genes and restoration of circadian rhythms, there is a lack of studies assessing the optimal timing of bioactive compound dosing to enhance their ability to regulate HTN.

## 6. Conclusions and Future Perspectives

The relevance of controlling BP is confirmed by the impact of HTN and CVD as a major problem within public health. Thus, novel approaches using pharmacological drugs are being investigated and, when HTN is still in development, natural bioactive compounds and nutritional interventions emerge as promising alternatives.

The study of the mechanisms of action of these bioactive molecules (e.g., phenolic compounds or bioactive peptides) is crucial but the consumption timing must be highlighted. In this sense, chrononutrition is a novel concept that gathers together biological rhythms along with diet and bioactive compounds. Thus, the effect of biological oscillations on BP regulation mechanisms, as well as on compound digestion and metabolization, should be considered in the development of new antihypertensive ingredients, since these factors could alter the effectiveness of these compounds against HTN. In addition, it is known that gut microbiota is also sensitive to biological rhythms [212] and, therefore, it is plausible to believe that these changes may also alter BP. However, little is known about these changes and their consequences and further research is needed.

Although several studies have been conducted following strategies based on chrononutrition, further studies must be carried out to fully understand the relation between bioactive compounds, rhythms and HTN.

## Data Availability

Not applicable.
